# Perceptions of nursing home staff, residents and relatives on the quality of person-centered care in Spain nursing homes across the COVID-19 Pandemic

**DOI:** 10.1186/s12877-026-07298-w

**Published:** 2026-05-07

**Authors:** Pau Moreno-Martin, Rosa Noell-Boix, Iván Sánchez-Martínez, Javier Jerez-Roig, Xavier Gómez-Batiste, Jaume-Miquel March-Amengual

**Affiliations:** 1 Research Group on Methodology, Methods, Models and Outcomes of Health and Social Sciences (M3O), Faculty of Health Sciences and Welfare, Centre for Health and Social Care Research (CESS), University of Vic—Central University of Catalonia (UVic-UCC), 08500 Barcelona, Vic Spain; 2 Institute for Research and Innovation in Life and Health Sciences in Central Catalonia (IRIS-CC), 08500 Barcelona, Vic Spain; 3 Department of Nursing, University of Girona, Girona, Spain; 4 Health and Healthcare Research Group, University of Girona, Girona, Spain; 5 Institute of Sport Science and Innovations, Lithuanian Sports University, 44221 Kaunas, Lithuania; 6 Chair of Palliative Care, Central Catalonia Chronicity Research Group (C3RG), Centre for Health and Social Care Research (CESS), Faculty of Medicine, University of Vic ‒ Central University of Catalonia, Barcelona, Vic Spain; 7 Chair of Medical Education, Centre for Health and Social Care Research (CESS), Faculty of Medicine, University of Vic—Central University of Catalonia (UVic-UCC), Vic, Barcelona Spain

**Keywords:** Person-centered care, Nursing home, Covid-19

## Abstract

**Background:**

Person-Centered Care (PCC) has become established as an essential model in geriatric care, promoting the dignity, autonomy, and well-being of residents. However, the COVID-19 pandemic posed an unprecedented challenge to its implementation in nursing homes (NH), affecting both the quality of care and human relationships. This study analyzes the perceptions of residents, relatives, professionals, and directors regarding PCC before, during, and after the pandemic in Catalonia (Spain).

**Methods:**

A multicenter cross-sectional study was conducted in 30 NH across the 10 healthcare regions of Catalonia. Validated PCC in Gerontology Services model questionnaires were administered to 335 participants, including residents, relatives, professionals, and directors. Each item was retrospectively assessed at three time points: before, during, and after the pandemic. Descriptive, staffing, and pandemic-related variables from the facilities were collected. Statistical analyses included descriptive statistics, nonparametric tests for group comparisons, and bivariate associations assessed using Spearman’s rank correlation coefficients.

**Results:**

During the COVID-19 pandemic, PCC scores dropped sharply—from 176 pre-pandemic to 136 during the crisis- before partially recovering to 171 post-pandemic, underscoring the substantial impact of the health emergency. Larger NHs and those with more months in “red code” experienced greater losses in PCC scores during the pandemic, while homes with a higher proportion of resident transfers showed smaller decreases. Additionally, NHs with a higher physician-to-resident ratio experienced larger decreases in PCC scores during the pandemic, while NHs that suffered greater losses in PCC scores tended to show greater post-pandemic recovery.

**Conclusion:**

This study confirms that PCC is particularly vulnerable during health crises such as the COVID-19 pandemic. The findings highlight the need for flexible organizational structures, adequate resources, and strong ethical guidance to ensure the sustainability of the PCC model and underscore the importance of contingency plans that maintain PCC without compromising residents’ safety, dignity, or autonomy.

**Trail registration:**

Not applicable.

## Introduction

The World Health Organization (WHO) has emphasized the urgent need to promote person-centered care (PCC) policies and practices, particularly in residential care settings, to address the complex challenges faced by older adults in their communities. This approach is essential not only to ensure the quality of care but also to uphold human rights and dignity throughout all stages of life [1]. Consequently, it has emerged as a central model in gerontological care, grounded in a biopsychosocial perspective that acknowledges each individual’s uniqueness and respects their needs, beliefs, and preferences [2, 3]. The model promotes active participation in care, enhancing physical, emotional, and social well-being [4–6], while also emphasizing the role of professionals and family members in placing the person at the center of decision-making [7].

Several studies have shown that implementing PCC in NHs is associated with more personalized care, greater resident satisfaction, and a reduction in neuropsychiatric symptoms such as agitation and depression. Moreover, it contributes to lowering perceived workload among care staff [3–5].

However, internationally, the COVID-19 pandemic posed a significant challenge to the continuity of PCC in NHs [7, 8]. Health restrictions, including isolation measures, reduced social contact, and limited physical interactions, directly challenged the ability to provide individualized care, impacting residents’ autonomy, social relationships, and emotional well-being [3, 8]. These challenges not only constrained the implementation of person-centered practices but also generated ethical tensions between protecting collective health and respecting individual needs, forcing geriatric care professionals to uphold fundamental PCC principles—such as autonomy and dignity—amid the pressures of a public health emergency [8, 9]. 

In Spain, research on person-centered care (PCC) remains limited, with most studies focusing on COVID-19’s impact on mortality and structural weaknesses in long-term care [10–12]. Factors linked to mortality outcomes—such as residential density, ownership, staffing shortages, and limited integration between health and social care—have been identified, but none of these factors have been specifically investigated in relation to PCC. Qualitative evidence from Lázaro et al [13]. highlights how the pandemic disrupted relational bonds essential for PCC, although team support provided resilience and trust. A recent international review further emphasized the scarcity of empirical studies on PCC continuity in NHs during COVID-19 [14]. To date, no empirical study has quantified changes in PCC during the COVID period or examined how structural and organizational NH variables influenced these changes.

The primary aim of this study was to evaluate how PCC changed during the COVID-19 pandemic period in long-term care facilities in Catalonia, based on the perceptions of residents, family members, staff, and facility directors collected at three key time points—before, during, and after the pandemic—as indicators of these changes. Furthermore, the study examined which structural and organizational characteristics of the facilities most strongly influenced these fluctuations, shedding light on factors that shaped the resilience and recovery of PCC during and after the health crisis.

## Methods

### Design

This multicenter observational retrospective study aimed to describe the perceptions of residents, family members, staff, and facility directors regarding the implementation of PCC in NH, and to evaluate these perceptions before, during, and after the COVID-19 pandemic. The study also explored the relationships between these perceptions and the physical and organizational characteristics of the facilities. This work is a substudy of the broader ResiCOVID-19 project and was conducted in 30 NH across the 10 health regions of Catalonia, Spain [15].

The study was conducted in accordance with the ethical principles outlined in the Declaration of Helsinki and was approved by the Ethics and Research Committee of the University of Vic – Central University of Catalonia (registration number: 181/2021). Written informed consent was obtained from all participants. Data collection took place between December 2021 and June 2022.

### Setting and sample

Catalonia has a total of 779 registered NH. For this study, facilities were stratified by three criteria: size (small, medium, large), ownership type (for-profit, non-profit, mixed), and health region (10 geographically defined areas based on socioeconomic and demographic factors). Within each stratum, simple random sampling was applied to select participating facilities. If a facility declined to participate, the next on the randomized list was contacted. A total of 30 NH were included, ensuring representation across all health regions and types of ownership. Within each facility, participants were recruited from four groups: residents, family members, direct care staff, and facility directors.

All facility directors of the selected NH were included. Each facility recruited 5–6 family members with a relative residing there before and during the pandemic; relatives of deceased residents were also eligible. All family participants were selected by the facility coordinator. Additionally, 5–6 professionals per facility who had worked there since before the pandemic were included, typically one nurse, two other healthcare professionals (e.g., psychologist, physiotherapist, or social worker), two geriatric care assistants, and occasionally another professional. Regarding residents, the original project included all individuals in small NH (≤ 50 residents) and a random 25% sample in larger homes; if a selected resident declined, the next on the randomized list was invited. There were no additional eligibility criteria beyond the willingness to participate and being selected in the sampling process. For this subset, only residents with sufficient cognitive capacity to complete the questionnaires were included, and those unable to respond adequately were excluded.

### Instruments

PCC was evaluated using the Person-Centered Care in Gerontology Services (PCC-G) questionnaire, a validated tool designed to assess the degree of person-centered care provided in long-term care settings from the perspective of key stakeholders [16, 17]. The PCC-G includes four tailored versions for staff, residents, relatives, and directors. Each version contains 22 Likert-type items (rated from 1 = totally disagree to 10 = totally agree), with the staff and directors versions including one additional item. The wording of each questionnaire is adapted to reflect the unique perspective of each respondent group. Items are organized into ten components aligned with the core dimensions of the PCC model.

To assess the impact of the COVID-19 pandemic, participants rated each item of the PCC-G at three distinct time points: retrospectively for the period before the pandemic [t0], during the pandemic [t1], and at the time of post-pandemic data collection [t2]. The impact of the pandemic was examined by calculating the difference between the score during the pandemic [t1] and the pre-pandemic score [t0], providing an indicator of the variation in PCC-G associated with COVID-19 (hereafter referred to as [t1–t0]). Similarly, subtracting the pre-pandemic score [t0] from the post-pandemic score [t2] yielded an indicator of PCC-G variation following the pandemic (hereafter referred to as [t2–t0]).

Age and sex of all participants were collected. Core structural and environmental characteristics of the residential facilities were systematically recorded. These included residents’ access to outdoor spaces within their buildings and whether these areas featured natural elements like gardens, trees, or plants. Data were also collected on the number of residents before the COVID-19 pandemic and at the time of data collection, the total bed capacity of each facility, and the proportion of shared rooms relative to the total number of rooms.

Institutional indicators describing the impact of the COVID-19 pandemic were also collected. These included the proportion of residents who died from COVID-19 and those who had been infected or recovered, both calculated relative to the total number of residents. Additional indicators captured the proportion of residents—again relative to the total resident population—who were transferred to their private homes, to other nursing or care facilities, or admitted to hospitals during the pandemic. Staff availability was assessed through the proportion of total sick leave days in relation to the number of full-time employees during the pandemic period [t1]. Operational difficulties were documented by recording the number of weeks each facility experienced shortages of protective or medical materials. The duration of emergency alert phases was also recorded, including the number of months each residence operated under “yellow code” and “red code” conditions, corresponding to moderate and high levels of epidemiological risk, respectively. Material shortages were measured as the number of weeks during which essential materials were reported as insufficient in the facility during the COVID-19 pandemic. Finally, staff confinement was measured as the total number of weeks during which professional staff remained confined within the facility on a 24-h basis as part of outbreak control measures.

Detailed staffing data were collected, including the number of personnel and their weekly working hours across professional categories. Four staff-to-resident ratios were calculated: total staff hours per resident, hours of technical staff per resident, hours of physicians per resident, and hours of geriatric care assistants per resident. Technical staff included physical and occupational therapists, social workers, and nurses not classified as geriatric care assistants, while physicians combined specialists and general practitioners, and geriatric care assistants encompassed both caregivers and auxiliary staff.

### Data collection

Prior to initiating data collection, selected NH were contacted via email and telephone. A video presentation was shared with each facility, outlining the study’s objectives, relevance, and participation procedures. A follow-up virtual meeting was held to address questions, coordinate logistics, and confirm the data collection schedule.

Data collection was conducted in person at each facility by two trained researchers, either physiotherapists or nurses, using tablet-based online questionnaires. Selected staff members completed their questionnaires in a single group session, with investigators present to assist and clarify any doubts. The same procedure was followed by family members. Residents were interviewed individually to ensure personalized and accurate responses. Facility directors received the questionnaire electronically and completed it independently at their convenience.

### Data analysis

A descriptive analysis was performed to present the absolute and relative frequencies of the categorical variables, and the mean and standard deviation (SD) of the quantitative variables. Normality was assessed using the Shapiro–Wilk test, and since all quantitative variables did not follow a normal distribution, nonparametric methods such as Mann–Whitney U tests were applied for the comparison of means. Bivariate associations were examined using Spearman's rank correlation coefficients. The Statistical Package for Social Sciences (SPSS) version 30 (SPSS Inc., Chicago, IL, USA) was used for all data analyses.

Data were analyzed at the NH level by aggregating responses from the different participant groups to obtain an overall mean representing the entire facility. This analytical approach provided a more objective and accurate estimate of the home’s average perception of PCC. By treating the NH as the unit of analysis—rather than individual respondents—the potential bias related to unequal group sizes was reduced, thereby improving the validity and contextual relevance of the results.

### Ethical considerations

Ethical approval for this study was obtained from the Ethics and Research Committee of the University of Vic – Central University of Catalonia (registration number: 181/2021), prior to the commencement of data collection. All procedures were conducted in accordance with the ethical standards of the Declaration of Helsinki. Written informed consent was obtained from all participants.

## Results

### Sociodemographic characteristics of participants

Participants across 30 NH in Catalonia, Spain, comprised 335 individuals, including 17.3% residents, 33.1% family members, 41.2% professionals, and 8.4% directors. Overall, 81.5% were female: 65.5% of residents, 78.4% of family members, 89.1% of professionals, and 89.3% of directors. The mean age for the total sample was 56.6 ± 18.1 years, with residents being the oldest group (83.7 ± 10.5), followed by family members (60.9 ± 10.9), directors (48.7 ± 9.6), and professionals (43.1 ± 10.7). Additional descriptive analyses can be found in Table 1.

**Table 1:** Sociodemographic Characteristics of Participants (Residents, Family Members, Staff, and Directors) Across 30 NH in Catalonia, Spain (*n* = 335)

	**n (%)/Mean** ± **SD**
Participants by group	
Residents	58 (17.3)
Relatives	111 (33.1)
Professionals	138 (41.2)
Directors	28 (8.4)
Sex	
Male	62 (18.5)
Female	273 (81.5)
Sex by groups	
Residents	
Male	20 (34.5)
Female	38 (65.5)
Relatives	
Male	24 (21.6)
Female	87 (78.4)
Professionals	
Male	15 (10.9)
Female	123 (89.1)
Directors	
Male	3 (10.7)
Female	25 (89.3)
Overall Age	56.6 ± 18.1
Age by group	
Residents	83.7 ± 10.5
Familiars	60.9 ± 10.9
Professionals	43.1 ± 10.7
Directors	48.7 ± 9.6

### Structural, environmental, and COVID-19-related indicators in NH

Among the 30 included NH, most were for-profit (83.3%) and provided residents with access to outdoor spaces (96.7%) and garden-like areas (70.0%). The mean number of residents decreased slightly from 84.7 ± 46.5 before the COVID-19 [t_0_] to 78.5 ± 39.6 at data collection [t_2_], with a total bed capacity of 86.7 ± 47.3 and 68.9 ± 28.3 of the facility's total rooms being shared rooms. COVID-19-related outcomes included a mean resident mortality of 16.2 ± 12.9 and infection in 54.6 ± 34.8. Regarding resident mobility, transfers to other nursing homes or care settings and hospitalizations involved, on average, 10.2 ± 22.6% and 13.4 ± 26.9% of residents, respectively, whereas transfers to private homes were the least frequent, affecting only 1.4 ± 3.9% of the resident population.

Weekly staffing ratios per resident averaged 2.8 ± 1.2 h for technical staff, 0.17 ± 0.1 h for physicians, 13.3 ± 7.4 h for care assistants, and 16.3 ± 8.0 total hours. Staff indicators included an average of 24.9 ± 26.2 sick leave days. Staff confinement due to outbreak control measures lasted 3.4 ± 9.8 weeks on average. Material shortages lasted an average of 4.1 ± 5.2 weeks. NH experienced an average of 2.6 ± 2.4 months under yellow code and 2.9 ± 2.2 months under red code. PCC-G scores decreased by 40.0 ± 15.9 points from [t_0_ to t_1_] and showed a partial recovery of 4.7 ± 7.6 points at [t_2_], remaining below the pre-pandemic value. These data are presented in Table 2.

**Table 2:** Structural, Environmental, and COVID-19-Related Institutional Indicators of the 30 NH in Catalonia, Spain (*n* = 30)

**Variable**	**n (%)/Mean** ± **SD**
Ownership (for profit)	25 (83.3)
Residents’ outdoor accessibility	29 (96.7)
Residents’ garden-like area accessibility	21 (70.0)
Number of residents [t_0_]	84.7 ± 46.5
Number of residents [t_2_]	78.5 ± 39.6
Total bed capacity	86.7 ± 47.3
Shared rooms per total rooms (%)	68.9 ± 28.3
Residents COVID-19 deaths (%) [t_1_]	16.2 ± 12.9
Residents COVID-19 cases (%) [t_1_]	54.6 ± 34.8
Residents transferred home (%) [t_1_]	1.4 ± 3.9
Residents transferred other NH or care settings (%) [t_1_]	10.2 ± 22.6
Residents hospitalized (%) [t_1_]	13.3 ± 26.9
Staff sick leave (days) [t_1_]	24.9 ± 26.2
Material shortage (weeks) [t_1_]	4.1 ± 5.2
Yellow code (months) [t_1_]	2.6 ± 2.4
Red code months (due to COVID-19 outbreak) [t_1_]	2.9 ± 2.2
Staff confinement (weeks) [t_1_]	3.4 ± 9.8
Technical staff hours per resident per week (ratio)	2.8 ± 1.2
Physician hours per resident per week (ratio)	0.2 ± 0.1
Care assistant hours per resident per week (ratio)	13.3 ± 7.4
Staff hours per resident per week (ratio)	16.3 ± 7.9
Decrease in PCC-G [t_1_–t_0_]	40.0 ± 15.9
Recovery in PCC-G [t_2_–t_0_]	4.7 ± 7.6

### Impact of COVID-19 on PCC

The mean PCC-G score for the entire sample at baseline pre-pandemic [t₀] was 175.7 ± 12.7. During the COVID-19 pandemic [t₁], the total score exhibited a significant reduction to 135.7 ± 21.3. At the post-pandemic assessment [t₂], the total score increased to 170.9 ± 3.1. Pairwise comparisons revealed that the differences between pre-pandemic and pandemic periods [t₁–t₀] and between pre-pandemic and post-pandemic periods [t₂–t₀] were statistically significant (*p* < 0.001 and *p* = 0.003, respectively).

**Fig. 1:**
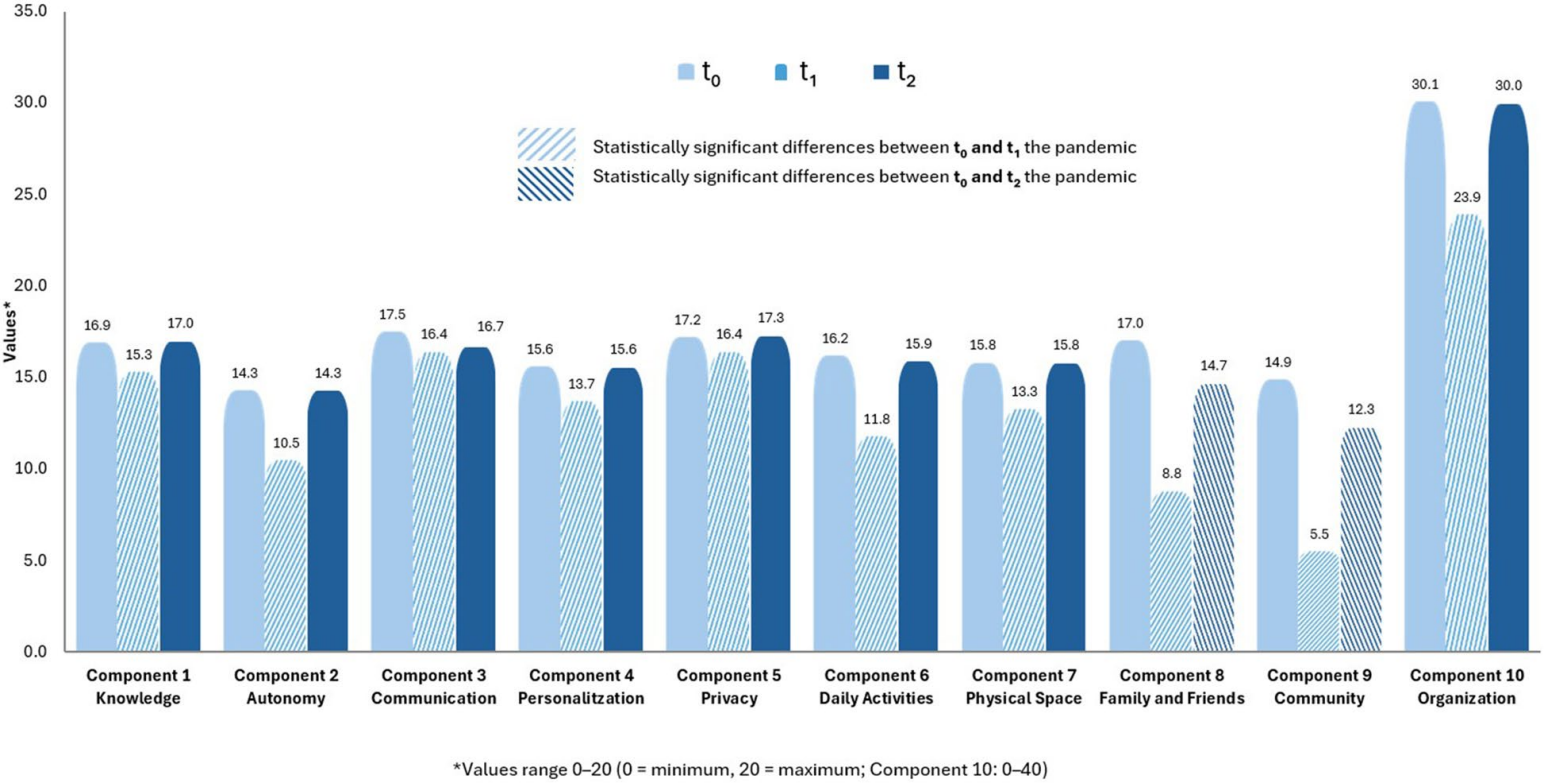
Values of the PCC-G dimensions before [t0], during [t1] and after [t2] the COVID-19 pandemic

Prior to the pandemic, the highest-scoring components were Communication (component 3) and Privacy (component 5), the only ones with scores above 17 (17.5 and 17.2, respectively). In contrast, Autonomy (component 2) and Community (component 9) had the lowest scores, both below 15 (14.3 and 14.9, respectively) (see Fig. 1).

All components showed a significant decrease (*p* < 0.001) in scores during the pandemic compared with the pre-pandemic period. By the time of data collection, most components had nearly returned to their pre-pandemic levels, as differences with initial scores were no longer statistically significant. However, Family and Friends (component 8) and Community (component 9) remained significantly lower than before the pandemic.

### Correlations between COVID-19–related changes in PCC-G and institutional variables

In Tables 3 and 4, Spearman correlations between institutional variables and COVID-19–related changes in Decrease in PCC-G [t_1_-t_0_] and Recovery in PCC-G [t_2_-t-_0_] are presented, with only statistically significant correlations shown, ordered by significance. Table 3 shows correlations with the Decrease in PCC-G due to COVID-19 [t_1_–t_0_]. Significant negative correlations of strong magnitude were observed for the number of residents (r = −0.654, *p* < 0.001) and total bed capacity (r = −0.676, *p* < 0.001). Red code months due to the COVID-19 outbreak (t_1_) also exhibited a strong significant negative correlation (r = −0.516, *p* = 0.006), while the Physician hours per resident per week ratio showed a moderate negative correlation (r = −0.429, *p* = 0.032). A significant positive correlation was found for the proportion of residents transferred to other NH or care settings during the pandemic (r = 0.496, *p* = 0.010).

**Table 3:** Correlation of variation in PCC-G associated with COVID19 [t_1_ – t_0_] and institutional variables according to Spearman’s test (*n* = 30 NH)

Variation in PCC-G associated with COVID19 (t_1_ – t_0_)				
**Variable**	**r**	**95% CI**		**P**
		**Lower**	**Upper**	
Number of residents [t_2_]	−0.695	−0.849	−0.431	< 0.001
Total bed capacity	−0.676	−0.837	−0.408	< 0.001
Number of residents [t_0_]	−0.654	−0.827	−0.368	< 0.001
Red code months (due to COVID-19 outbreak) [t_1_]	−0.516	−0.754	−0.157	0.006
Residents transferred other NH or care settings (%) [t_1_]	0.496	0.123	0.746	0.010
Recovery in PCC-G [t_2_ – t_0_]	0.463	0.112	0.711	0.010
Physician hours per resident per week (ratio)	−0.429	−0.711	−0.028	0.032

**Table 4:** Correlation of variation in PCC-G following the pandemic [t_2_ – t_0_] and institutional variables according to Spearman’s test (*n* = 30 NH)

**Variation in PCC-G following the pandemic (t**_**2**_** – t**_**0**_)				
**Variable**	**r**	**95% CI**		**p**
		**Lower**	**Upper**	
Decrease in PCC-G [t_1_ – t_0_]	0.463	0.112	0.711	0.010
Physician hours per resident per week (ratio)	−0.460	−0.730	−0.067	0.021

Table 4 presents correlations with the Recovery in PCC-G after COVID-19 [t_2_–t_0_]. Recovery was positively correlated with the Decrease in PCC-G during the pandemic (t_1_–t_0_) (r = 0.463, *p* < 0.010) and negatively correlated with the Physician hours per resident per week (ratio), also with moderate strength (r = −0.460, *p* = 0.021).

## Discussion

The primary aim of this study was to evaluate how PCC fluctuated throughout the COVID-19 pandemic in Catalonia’s NH. We considered the perceptions of residents, family members, staff, and facility directors as an indicator of these variations. In addition, we explored which structural and organizational characteristics of the facilities had the greatest influence on these fluctuations. PCC scores declined significantly during the pandemic, especially in larger and more medically staffed NH, while facilities with more resident transfers showed greater resilience, and those most affected tended to recover more strongly afterward.

The study sample, comprising residents, family members, staff, and facility directors, aligns with the composition reported in prior research within residential care contexts [3, 17]. Consistent with existing literature, the COVID-19 pandemic profoundly affected all ten components of PCC [18]. During the pandemic, prioritizing residents’ safety over autonomy and dignity created profound ethical dilemmas [8]. Emergency measures—such as isolation, visit restrictions, use of personal protective equipment, and spatial reorganization—disrupted individualized care and negatively affected physical and emotional well-being [8, 19–22]. In Catalonia, strategies like residence classification by isolation capacity, staff redistribution, creation of alternative spaces, and digital tools to maintain family contact sought to balance infection control with person-centered care, yet underscored persistent ethical tensions [23, 24]. 

From an organizational perspective, it was observed that larger facilities and those that remained longer in “red code” status—i.e., under intensified restrictions due to active COVID-19 outbreaks—experienced a greater decline in the perception of PCC, consistent with previous studies in the literature [7, 18]. These observations underscore how specific institutional characteristics amplified the pandemic’s impact on care quality and PCC implementation [18]. In accordance with previous research [25] reported that smaller, more person-centered care models, such as Green Houses, achieved superior outcomes in infection control and resident well-being. Furthermore, higher population density and shared rooms have been linked to increased rates of respiratory infections and mortality even prior to the COVID-19 pandemic, highlighting the critical role of facility design and resident density in supporting both infection prevention and quality of care [25–27].

However, certain organizational decisions mitigated these effects. Facilities that implemented a higher number of resident transfers during the pandemic experienced a smaller decline in PCC quality. Planned transfers that respect individual preferences and maintain continuity of care can reduce anxiety and improve care quality [28]. In Catalonia, these transfers were carried out as a strategy to contain the spread of the virus [29]. Furthermore, the magnitude of post-pandemic recovery in PCC was positively associated with the severity of the initial decline, indicating that facilities most affected during the pandemic tended to show the greatest improvements. This pattern may reflect the influence of targeted recovery efforts or adaptive strategies, in some cases approaching or surpassing pre-pandemic levels of perceived care quality [3].

Paradoxically, a higher medical staffing level was associated with a poorer perception of PCC, both during the pandemic and in the recovery phase. This counterintuitive finding may be explained by the implementation of more rigid protocols, increased medicalization of the facilities, and a focus on survival, which came at the expense of care quality and the promotion of residents’ dignity and autonomy, highlighting the inherent dilemma between medical priorities and person-centered care under crisis conditions [18, 30]. In this context, increases in staffing ratios focused only on medical roles may reinforce a biomedical model; while essential for clinical safety, they can conflict with the holistic and psychosocial support central to PCC, emphasizing that professional balance matters as much as staffing levels [8].

At the level of social relationships, the pandemic profoundly disrupted the connections between residents, families, and staff. The “community” and “family and friends” components—reflecting residents’ connection to the broader social environment and their close personal relationships, respectively—were the only aspects that did not recover significantly, highlighting the persistent impact of restrictions on social contact. These findings may reflect the greater structural vulnerability of PCC dimensions that rely heavily on interpersonal interaction, relational continuity, and shared decision-making. During health crises, organizational priorities tend to shift toward infection control, risk management, and operational safety, which can unintentionally displace practices centered on autonomy, social participation, and family involvement. Previous research has highlighted how pandemic-related restrictions generated ethical tensions between collective protection and individual needs, often leading to reduced opportunities for relational care and participation in decision-making [8, 9, 18]. Moreover, isolation measures and visitation restrictions have been shown to disrupt social relationships and individualized care processes in long-term care settings, further compromising relational aspects of PCC [19, 21]. Although some facilities demonstrated adaptability and creativity, implementing strategies to promote communication and emotional expression [16, 17, 19, 21], these measures were insufficient to fully compensate for the absence of in-person interaction.

Overall, these findings align with international evidence indicating that the resilience of PCC during the pandemic was highly dependent on institutional context. In Sweden, facilities specializing in dementia care with strong leadership were able to sustain higher levels of PCC [3]. In the United States centers with more comprehensive PCC implementation experienced significantly lower rates of infection, hospitalization, and mortality, highlighting the clinical as well as emotional benefits of high-quality care [31]. In Spain, although the overall perceptions of individualized care remained largely stable, specific aspects related to emotional expression improved after the pandemic [32]. Taken together, these studies emphasize that factors such as facility type, leadership strength, and organizational culture are key determinants of PCC resilience, with important implications for both resident well-being and clinical outcomes.

Within this context, two primary categories of ethical dilemmas emerged: access to care (e.g., triage, ICU admission, vaccination) and infection control decisions (e.g., isolation versus autonomy), with potential solutions including more flexible protocols, enhanced communication, caregiver support, and the ethical use of technology [33]. The scientific literature consistently emphasizes that emergency policies should uphold principles that preserve and support PCC. In Spain, Amblàs-Novellas and Gómez-Batiste [34] proposed specific clinical and ethical recommendations for decision-making in residential care, highlighting the importance of proportionality, individualized care, and respect for autonomy in high-pressure situations, and illustrating the ongoing effort to reconcile health protection with PCC [34].

These findings underscore the critical need for contingency planning that maintains the continuity of PCC during health emergencies while safeguarding residents’ safety, dignity, and autonomy [4, 17]. Beyond operational preparedness, the results highlight the importance of embedding PCC principles into the design, regulation, and oversight of residential care systems [35]. Policies that support smaller, more adaptable organizational structures, adequate interdiscplinary staffing ratios, and fair working conditions not only enhance the resilience of long-term care facilities during crises but also promote sustained care quality under normal conditions [36]. Moreover, integrating PCC indicators into quality monitoring frameworks and contingency plans can serve as a benchmark for ethical and effective care, ensuring that residents’ rights, dignity, and well-being remain central even in high-pressure or emergency scenarios [14].

This study has several limitations. Data on PCC before and during the pandemic were collected retrospectively, introducing potential recall bias, and institutional variables were measured only once post-pandemic, limiting longitudinal insights. The observational design precludes causal inferences between organizational factors and PCC outcomes. The resident sample was small and less representative due to high COVID-19 mortality, exclusion of cognitively impaired individuals, and recruitment challenges while facilities were still recovering. Recruitment challenges were further compounded by the fact that, at the time of data collection, facilities were still recovering from the pandemic and remained highly vulnerable. Efforts to minimize losses further constrained sample size, reducing statistical power and preventing multivariate analyses to control confounders. Although aggregating PCC scores at the facility level facilitates comparisons with organizational variables, this approach may mask within-facility heterogeneity in perceptions among residents, relatives, frontline staff, and management, whose evaluations of PCC are known to differ [17, 37]. While the study identifies structural and ethical dimensions influencing PCC, future research should adopt longitudinal designs, include cognitively impaired populations, and explore factors such as leadership, staff resilience, and digital tools to support relational care and mitigate isolation.

Despite these limitations, the study has several notable strengths, including being the first to assess PCC before, during, and after the COVID-19 pandemic in a representative sample of thirty nursing homes across Catalonia’s ten health regions. A key strength of the study is its use of the PCC in Gerontology Services model, which captures and systematically integrates the perspectives of directors, staff, residents, and families—whose assessments of PCC often differ [17, 37] —while being validated and adapted for each group, thereby providing a comprehensive evaluation of pandemic-related changes in PCC and enhancing both internal validity and international comparability [16, 17]. The use of the PCC-G instrument, validated and adapted for each group, strengthens internal validity and enhances international comparability of the findings. Moreover, to date, this study is the first to explore the relationship between structural and organizational characteristics—such as facility size, duration in “red code” status, proportion of shared rooms, and perceived PCC quality—during the pandemic.

## Conclusion

This study confirms that PCC is particularly vulnerable in emergency health crises, such as the COVID-19 pandemic. The perceived quality of care declined significantly during the crisis, with only partial recovery observed in the post-pandemic period. Larger and more medically staffed facilities experienced greater declines, while smaller, more flexible NH demonstrated higher resilience. The findings highlight the need for adaptable organizational structures, adequate resources, and strong ethical reflection to ensure the sustainability of the PCC model. For future health emergencies, these results underscore the importance of contingency plans that preserve PCC without compromising residents’ safety, dignity, or autonomy.

## Data Availability

The datasets used or analyzed during the current study are available from the corresponding author upon reasonable request.

## Declarations

Ethical approval (registration number 181/2021) was obtained from the Ethics and Research Committee of the University of Vic– Central University of Catalonia prior to the commencement of data collection. Signed informed consent was obtained from the residents, relatives, professionals and directors. All the methods and procedures carried out in this study were in accordance with relevant guidelines and regulations.
